# Distance learning in clinical medical education amid COVID-19 pandemic in Jordan: current situation, challenges, and perspectives

**DOI:** 10.1186/s12909-020-02257-4

**Published:** 2020-10-02

**Authors:** Mahmoud Al-Balas, Hasan Ibrahim Al-Balas, Hatim M. Jaber, Khaled Obeidat, Hamzeh Al-Balas, Emad A. Aborajooh, Raed Al-Taher, Bayan Al-Balas

**Affiliations:** 1 General and Breast Surgery, Department of General and Special Surgery, Faculty of Medicine, Hashemite University, Irbid-Amman Street, Al Husn, P.O. Box 3, Irbid, 21510 Jordan; 2 Otorhinolaryngology, Faculty of Medicine, Yarmouk University, Irbid, Jordan; 3 Community Medicine, Faculty of Medicine, Al-Balqa Applied University, Salt, Jordan; 4 Transplant and Hepatopancreaticobiliary Surgery, Department of General Surgery, Faculty of Medicine, Jordan University of Science and Technology, Irbid, Jordan; 5 General and Gastrointestinal Surgery, Department of General and Special Surgery, Faculty of Medicine, Hashemite University, Zarqa, Jordan; 6 General and Gastrointestinal Surgery, Department of General Surgery, Faculty of Medicine, Mutah University, Mu’tah, Jordan; 7 Pediatric surgery, Department of general surgery, Faculty of Medicine, Jordan University, Irbid, Jordan; 8 Yarmouk University, Irbid, Jordan

**Keywords:** COVID-19, Distance learning, E-learning, Medical education

## Abstract

**Background:**

As COVID-19 has been declared as a pandemic disease by the WHO on March 11th, 2020, the global incidence of COVID-19 disease increased dramatically. In response to the COVID-19 situation, Jordan announced the emergency state on the 19th of March, followed by the curfew on 21 March. All educational institutions have been closed as well as educational activities including clinical medical education have been suspended on the 15th of March. As a result, Distance E-learning emerged as a new method of teaching to maintain the continuity of medical education during the COVID-19 pandemic related closure of educational institutions. Distance E-Learning is defined as using computer technology to deliver training, including technology-supported learning either online, offline, or both. Before this period, distance learning was not considered in Jordanian universities as a modality for education. This study aims to explore the situation of distance E-learning among medical students during their clinical years and to identify possible challenges, limitations, satisfaction as well as perspectives for this approach to learning.

**Methods:**

This cross-sectional study is based on a questionnaire that was designed and delivered to medical students in their clinical years. For this study, the estimated sample size (*n* = 588) is derived from the online Raosoft sample size calculator.

**Results:**

A total of 652 students have completed the questionnaire, among them, 538 students (82.5%) have participated in distance learning in their medical schools amid COVID-19 pandemic. The overall satisfaction rate in medical distance learning was 26.8%, and it was significantly higher in students with previous experience in distance learning in their medical schools as well as when instructors were actively participating in learning sessions, using multimedia and devoting adequate time for their sessions. The delivery of educational material using synchronous live streaming sessions represented the major modality of teaching and Internet streaming quality and coverage was the main challenge that was reported by 69.1% of students.

**Conclusion:**

With advances in technologies and social media, distance learning is a new and rapidly growing approach for undergraduate, postgraduate, and health care providers. It may represent an optimal solution to maintain learning processes in exceptional and emergency situations such as COVID-19 pandemic. Technical and infrastructural resources reported as a major challenge for implementing distance learning, so understanding technological, financial, institutional, educators, and student barriers are essential for the successful implementation of distance learning in medical education.

## Background

Covid-19 has been declared as a pandemic disease by the WHO on March 11th, 2020. The disease started in Wuhan province in China in late December 2019. Since that time, the global incidence of COVID-19 disease has increased dramatically.

In early March 2020; the first diagnosed case of COVID-19 disease had been reported in Jordan for a Jordanian citizen who had returned from Italy 2 weeks before Jordan has declared quarantine for Jordanians returning from outside. By the end of March, the total number of infected persons was 274 cases. The number of diagnosed cases showed a steady slow increase, and by the end of April, a total of 453 diagnosed cases with 8 deaths were reported.

On the 19th of March, the emergency state was announced in Jordan followed by the curfew on 21 March. Similar to other sectors, the educational sector has been affected by this pandemic situation. All educational institutions have been closed as well as educational activities have been suspended on the 15th of March. As part of that, the six medical schools in Jordan in stopped all their teaching and training activities. Distance E-learning has emerged as a new method of teaching to maintain the continuity of medical education during the COVID-19 pandemic related closure of educational institutions.

Medical education programs in Jordan are six-year programs, in which clinical medical years are the last 3 years in the curriculum. During clinical education, students receive both in-class theoretical lectures and seminars, and in-hospital clinical rotations. Before the era of COVID-19, distance e-learning was not adopted as a modality of teaching within medical schools.

Distance E-learning in medical education may represent a suitable alternative to traditional learning to deliver high-quality education. The availability of essential infrastructures and efficient institutional strategies represent a major challenge for integrating distance learning in medical education [1]. Even blended education (i.e. distance and on-campus) is well adopted in different word countries, the effect of distance electronic learning is likely to be revolutionary specially in low-middle income countries.

Distance E-Learning is defined as using computer technology to deliver training, including technology-supported learning either online, offline, or both [2]. It is aimed at the effective construction of knowledge regarding individual experience, practice, and knowledge of the learners and students [3]. Internet-based learning, computer-based learning, virtual classrooms, and digital collaboration all represent different types of e-learning.

There are 2 modes of E-learning: distance learning and computer-assisted interaction (CAI). Moore et al. defined distance E-learning as providing access to learning for those who are geographically remote from the instructor [2], while CAI is an interactive technique whereby instructional material is presented by and a computer, and students’ progress is monitored and evaluated during this process.

Distance E-learning has been proved as an efficient modality of learning in different educational and governmental studies [4–6]. Data from the Institute of Educational Studies in Canada showed that learners revealed a more active attitude in learning when various methods such as electronic books and on-line articles were implemented in the teaching process [4, 7].

Anderson et al. in their paper have described three generations of distance education pedagogy. These generations are Cognitive-behaviourism, Constructivism, and Connectivism. Each one of these generations has distinguished utilized technology, learning activities, learner and content granularity, evaluation modality, scalability and instructor role. These generations have developed in concordance with available technologies. They stated that no single modality has provided all answers, and each of these generations has built on achievements of previous generation [8].

According to the 2030 Sustainable Development Goals (SDGs) for health including good health well-being and quality of education, effective and affordable educational strategies need to be addressed critically especially in low and middle-income countries (LMIC) [9]. Adopting distance e-learning in different fields of knowledge in low and middle-income countries (e.g. Jordan) can add a great benefit to achieve 2030 SDGs.

This study aims to explore the situation of distance e-learning among medical students during their clinical years and to identify possible challenges, limitations, satisfaction as well as their perspectives for this the approach of learning. To the best of our knowledge, no published study discussing the current situation of distance e-learning among Jordanian medical students in their clinical years is available. Students’ satisfactions, limitations, and perspectives have been addressed in our study as well.

## Methods

This is a cross sectional study based on a questionnaire of 23 questions that was designed by a team of faculty members from all participating medical schools in Jordan. Participating students are medical students in their clinical medical years (i.e. 4th, 5th and 6th years) from the all medical schools (i.e. University of Jordan, Jordan University of Science and Technology, Yarmouk University, Hashemite University, Mutah University, and Al-Balqa’a University)).

The questionnaire was distributed on May 5th, 2020 using previously created students Facebook and WhatsApp groups that were adopted by medical schools for communication with their students. Questions were in two formats, multiple-choice and rating questions on a five-point scale (Likert scale). For simplifying statistical analysis, the five categories were regrouped into three categories, and were labeled as agree, neutral and disagree.

These questions were mainly about student’s demographics (age, gender, academic years and medical school), prior and current experience with distance learning, available technologies, distance learning benefits, drawbacks, their instructors’ influence, challenges, attitudes towards the effectiveness of distance learning in medical education, and their perceptions about the future of distance learning in medical education.

For this study, the estimated sample size is derived from the online Raosoft sample size calculator [10]. The sample size was calculated based on a response rate of 50%, a confidence interval of 99%, and a margin of error of 5%, with a total 4th, 5th, and 6th medical student population around 5147 (i.e. University of Jordan (*n* = 1155), Jordan University of Science and Technology (*n* = 1525), Hashemite University (*n* = 694), Yarmouk University (*n* = 614), Mutah University (*n* = 910) and AlBalqa’a University (*n* = 249)), the largest required sample size is 588. Accordingly, this study included a convenient sample of 652 students who are currently enrolled in their clinical years.

Objectives and goals were explained at the beginning of the questionnaire to all participating students, and their enrollment was after they consent to participate in the study. Data were analyzed using the statistical package for the social sciences version 23 (SPSS Inc., Chicago, IL) statistical software. The analyses included descriptive statistics and chi-square test. A *P* < 0.05 was considered statistically significant.

## Results

The mean age of participants was 22.7 years (range: 21–28). As illustrated in Table 1, among the 652 students who completed their questionnaire, 382 (58.6%) were female. Other demographics are explained in Table 1. Two hundred and sixty-two students (40.2%) declared their enrollment in at least one distance learning course before or during the COVID-19 pandemic that is not part of their medical school curriculum while 149 students (22.9%) have participated in a distance learning course in their universities previously. Amid the COVID-19 pandemic, the majority of the student (*n* = 538; 82.5%) have participated in distance learning in their medical schools. Further analysis of the non-participating students showed that 6th-year students were the least to be involved in distance learning as the majority of them have completed their academic courses before the COVID-19 curfew. Among the 538 students, smartphones were the most commonly used single device in e-learning (35.9%) followed by computers either laptops or desktops (14.5%). Two hundred and sixty seven students (49.6%) utilized multiple devices to access their learning sessions.

**Table 1:** Participating students’ characteristics

		Number (%)
Age	Mean: 22.7 years (range: 21–28)	
Gender	Male	270 (41.4%)
	Female	382 (58.6%)
Clinical academic year	Fourth year	243 (37.3%)
	Fifth year	228 (34.9%)
	Sixth year	181 (27.8%)
Medical school	University of Jordan	96 (14.7%)
	Hashemite University	122 (18.7%)
	Yarmouk University	113 (17.3%)
	Mutah University	155 (23.8%)
	Al- Balqa’ Applied University	43 (6.6%)
	Jordan University of Science and Technology	123 (18.9%)
Previous enrollment in distance learning not related of university curriculum	Yes	262 (40.2%)
	No	390 (59.8%)
Previous enrollment in distance learning related to university curriculum	Yes	149 (22.9%)
	No	503 (77.1%)
Current enrollment in distance learning amid COVID-19	Yes	538 (82.5%)
	No	114 (17.5%)

According to the participating students, the delivery of educational material using synchronous live streaming sessions represented the major modality of teaching, in which 80.9% of students were engaged. Only prerecorded sessions and a mix of live and pre-recorded lessons were used in 11.2 and 8% respectively.

Various platforms and applications had been implemented in distance learning including ZOOM, Microsoft Teams, WhatsApp groups, Facebook groups, YouTube channels, Moodle, and Skype. While most students reported using multiple platforms in their learning (64.7%), ZOOM was the most commonly used single platform in delivering educational sessions (35.3%).

Regarding reported benefits, drawbacks, and challenges of distance learning, 55.9% reported having multiple advantages including time-saving, flexibility of class, improved interaction with instructors and classmates. A minority of students (5%) reported no benefits in comparison to traditional learning. The main drawbacks were the low quality of teaching reported by (48.3%) of responders and poor interaction with instructors reported by (62.1%) of responders. Internet streaming quality and coverage was the main challenge that was reported by 372 students (69.1%). A summary of reported benefits, drawbacks, and challenges is summarized in Table 2.

**Table 2:** Distance e-learning reported benefits, drawbacks and challenges (*n* = 538)

		Number of students (%)
Benefits	Time saving	425 (79%)
	Flexibility of class time	343 (63.8%)
	Better instruction	59 (11%)
	Improved learning	73 (13.6%)
	Better interaction instructor	73 (13.6%)
	Better interaction with classmates	18 (3.3%)
	No benefits	27 (5%)
Drawbacks	Poor instruction	260 (48.3%)
	Poor interaction with instructors	334 (62.1%)
	Poor interaction with classmates	308 (57.2%)
	No drawbacks	91 (16.9%)
Challenges	Poor internet coverage	372 (69.1%)
	Limitation in internet data packages	205 (38.1%)
	Lacking suitable devices	65 (12.1%)
	Variation in educational platforms	205 (38.1%)
	No challenges	40 (7.4%)

**Fig. 1:**
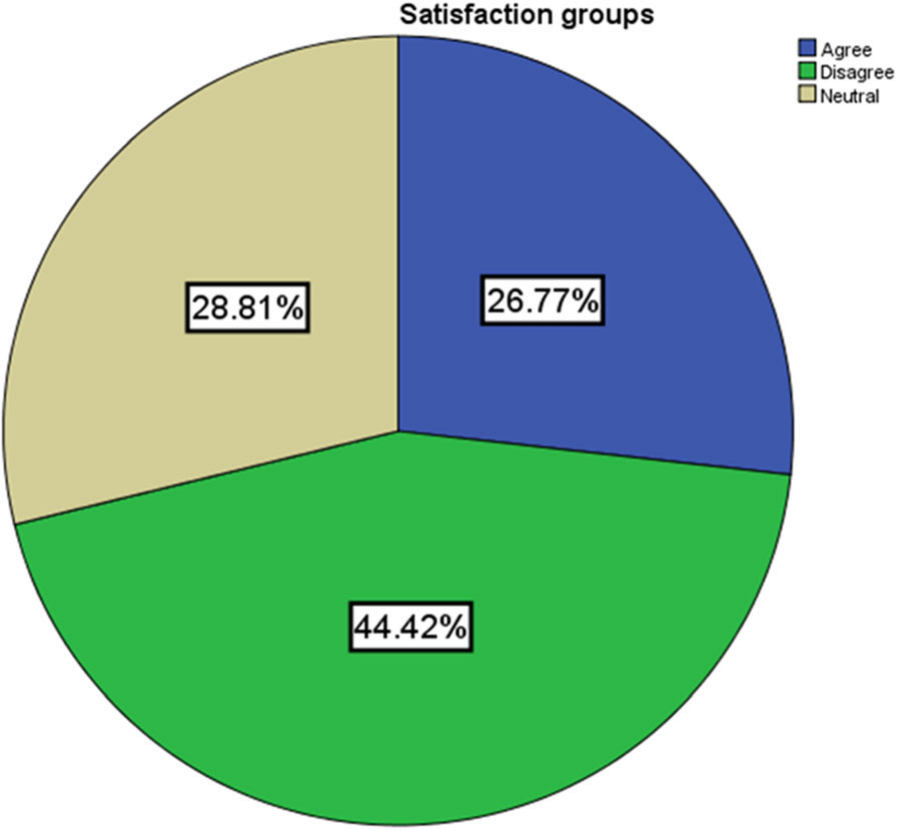
Students’ satisfaction with distance e-learning during clinical medical years

Based on students’ opinions regarding instructors’ role and performance in distance learning, 64.3% of students had agreed that instructors were actively participating in their discussions and 78.3% of students admitted that instructors approached them using multimedia to achieve desired course objectives. The majority of students reported an efficient response to their inquiries from instructors (i.e. 86.6% reported response in less than 48 h). On the other hand, the time dedicated to distance learning sessions was not adequate according to 26.5% of students. Overall, only 144 students (26.77%) were satisfied with their experience in medical distance learning (Fig. 1).

Factors that were significantly linked to the level of satisfaction includes students previous experience in distance learning within universities, instructors’ role, using multimedia and devoting adequate time for educational sessions. On the other hand, students’ academic level or previous enrollment in distance learning courses out of the university curriculum was not related to the level of satisfaction (Table 3).

**Table 3:** correlation between students’ level of satisfaction and different variables

		Level of satisfaction			
	Academic year	Satisfied	Neutral	Not satisfied	*P-* value
Academic year	4th	51 (9.5%)	58 (10.8%)	90 (16.7%)	0.444
	5th	61 (11.3%)	51 (9.5%)	91 (16.9%)	
	6th	32 (5.9%)	46 (8.6%)	58 (10.8%)	
Previous enrollment in distance learning not related of university curriculum	No	73 (13.6%)	98 (18.2%)	145 (27%)	0.064
	Yes	71 (13.2%)	57 (10.6%)	94 (17.5%)	
Previous enrollment in distance learning related to university curriculum	No	96 (17.8%)	117 (21.7%)	191 (35.5%)	0.015*
	Yes	48 (8.9%)	38 (7.1%)	48 (8.9%)	
Instructors actively participate in discussion	Agree	126 (23.4%)	116 (21.6%)	103 (19.1%)	< 0.0001**
	Neutral	17 (12.7%)	29 (5.4%)	88 (16.4%)	
	Disagree	1 (2.3%)	10 (1.9%)	48 (8.9%)	
Instructor use multimedia in teaching session	Agree	130 (24.2%)	131 (24.3%)	160 (29.7%)	< 0.0001**
	Disagree	14 (2.6%)	24 (4.5%)	79 (14.7%)	
The time dedicated for the E-learning courses is adequate	Agree	111 (20.6%)	73 (13.6%)	48 (9%)	< 0.0001**
	Neutral	28 (5.2%)	61 (11.3%)	74 (13.8%)	
	Disagree	5 (0.9%)	21 (3.9%)	117 (21.7%)	
Future course preferences	Entirely E-learning	21 (3.9%)	6 (1.1%)	1 (0.2%)	< 0.0001**
	Blended approach	120 (22.3%)	129 (24%)	157 (29.2%)	
	Traditional learning	3 (0.6%)	20 (3.7%)	81 (15.1%)	

* Significant

** Strongly significant

### Students future perspectives regarding distance e-learning

Based on students’ perspectives, implementing distance e-learning in medical education is challenging (Table 2); 65.8% of students think that preference of traditional approach by faculty members will be the main barrier and 46.8% believe that lack of cooperation from instructors will affect this approach. Poor financial capacity of students might also impede distance learning according to 297 students (55.2%). Besides that, 55.2% of students predict that lack of commitment to distance learning courses by students might discourage medical schools from adopting it in their curricula.

More than half of students (57.2%) admit that young staff members are more willing to participate in distance e-learning while 12.1% disagreed with that. According to 281 students (52.2%), distance e-learning can replace traditional class learning in delivering theoretical knowledge while 423 students (78.6%) replied that distance e-learning will represent a major challenge to acquire adequate clinical medical skills. According to 406 students (75.5%), blended approach (A mix of traditional and E-learning classes) is the preferred way to deliver medical education in the future, and adopting future distance learning was significantly related to the degree of overall satisfaction.

## Discussion

This study aimed to evaluate clinical medical students’ experiences in computer mediated distance e-learning, which is considered a newly adopted approach in Jordanian medical schools. Distance e-learning has emerged as a new method of teaching to maintain the continuity of medical education during the COVID-19 pandemic.

In this study, we explored student’s opinions toward major challenges they faced during their new experience of learning, limitations, faculty staff performance, overall satisfaction as well as future perspectives.

Traditional (i.e. face to face) teaching is considered an essential long-standing approach in medical education [11]. As traditional approaches in medical education are facing increased challenges because of the increase in clinical demands and reduction in available time [12], a shift in traditional medical education practice to online, distance, or electronic learning has been noticed in the last few decades [13].

In 2011, almost 30% of students were enrolled in distance learning during their undergraduate bachelor’s degree according to the US national center for educational statistics [14], but actually this is not the case in undergraduate education in Jordan. Distance e-learning has been reported to provide easier and more effective access to a wider variety and greater quantity of information, as well, learning delivery allows a personalized approach in learning, so students have more control over the educational content, learning sequence and time [15–17]. Another advantage of e-learning is the easier and faster update and distribution of the educational content in comparison to printed references. In this study, main reported advantages were saving time and flexibility of classes as being reported by 79 and 63.8% respectively.

Implementation of distance e-learning in medical education is challenging, especially in low-middle income countries. Barrier against adopting distance e-learning can be divided into three main levels; (1) technology/infrastructure barriers (2) institutional/educators’ barriers (3) student barriers. The lack of infrastructure, technology, internet access, and poor quality of internet services are examples of barriers that impact both learners and faculty members [18, 19], and this was demonstrated in participants reported challenges (Table 2).

Another important challenge against distance learning is the reluctance and avoidance of educators to engage in new technologies and applications because of their limited knowledge or lacking proper training in these fields [20]. As institutional support is essential for the success of distance learning, and institutional strategy should be designed to facilitate the implementation of key skills and the adoption of methodologies by faculty [18].

In this study, the overall medical students’ early experience in distance learning is unsatisfactory. Previous experience in distance learning was significantly associated with higher satisfaction among our students. Despite their low level of satisfaction, they expect that distance e-learning can partially replace the traditional method in delivering theoretical but not clinical skills. They believe that a blended approach (traditional and e-learning) will be the most suitable for future medical training.

### Strengths and limitations

Based on our review, this is the first paper discussing clinical medical distance e-learning in Jordan. We have involved medical students during their clinical years in all Jordanian medical universities. Major limitations for this study was the inability to measure educational outcomes linked to distance e-learning and comparing them to traditional learning. Further studies are required to address educational outcomes as well as faculty members’ perceptions and opinions towards distance e-learning.

## Recommendations for future practice

Based on the results, medical institutions in Jordan need to address previously mentioned challenges in near future to optimize the experience of distance e-learning among their students. It is mandatory to collaborate with telecommunication companies to provide students and instructors with high-quality internet coverage with affordable costs, also to improve instructors’ skills in using technology in their distance learning. The need to establish a unified educational platform for all medical schools might be the optimal solution to overcome the variation between students’ satisfaction in distance learning secondary to variation in instructors’ performance.

## Conclusion

With advances in technologies and social media, distance learning is a new and rapidly growing approach for undergraduate, postgraduate, and health care providers. Regardless of reported benefits, medical students preferred the blended approach in teaching as distance learning represented a major challenge to acquire adequate clinical medical skills. Satisfaction in distance learning is strongly linked to students’ prior experience in distance learning as well as instructors’ experiences and interactions. Technical and infrastructural resources reported as a major challenge for implementing distance learning, so understanding technological, financial, institutional, educators, and student barriers are essential for the successful implementation of distance learning in medical education.

## Data Availability

All data and supplementary material are available upon request from the corresponding author.

## Abbreviations

WHO: World Health Organization
COVID-19: Coronavirus disease 2019
SDGs: Sustainable Development Goals
LMIC: Low and middle-income countries
