# Correction to: Distance learning in clinical medical education amid COVID-19 pandemic in Jordan: current situation, challenges, and perspectives

**DOI:** 10.1186/s12909-020-02428-3

**Published:** 2020-12-16

**Authors:** Mahmoud Al-Balas, Hasan Ibrahim Al-Balas, Hatim M. Jaber, Khaled Obeidat, Hamzeh Al-Balas, Emad A. Aborajooh, Raed Al-Taher, Bayan Al-Balas

**Affiliations:** 1 Department of General and Special Surgery, Faculty of Medicine, Hashemite University, Zarqa, Jordan; 2 Otorhinolaryngology, Faculty of Medicine, Yarmouk University, Irbid, Jordan; 3 Community Medicine, Faculty of Medicine, Al-Balqa Applied University, Salt, Jordan; 4 Department of General Surgery, Faculty of Medicine, Transplant and Hepatopancreaticobiliary Surgery, Jordan University of Science and Technology, Irbid, Jordan; 5 Department of General and Special Surgery, Faculty of Medicine, General and Gastrointestinal Surgery, Hashemite University, Zarqa, Jordan; 6 Department of General Surgery, Faculty of Medicine, General and Gastrointestinal Surgery, Mutah University, Mu’tah, Jordan; 7 Pediatric Surgery, Department of General Surgery, Faculty of Medicine, Jordan University, Irbid, Jordan; 8 Yarmouk University, Irbid, Jordan

**Correction to: BMC Med Educ 20, 341 (2020)**

**https://doi.org/10.1186/s12909-020-02257-4**

Following publication of the original article [1], the corresponding author Mahmoud Al-Balas would like to correct his affiliation. In this Correction, correct and incorrect versions are shown.

Incorrect: General and Breast Surgery, Department of General and Special Surgery, Faculty of Medicine, Hashemite University, Irbid-Amman Street, Al Husn, P.O.Box 3, Irbid 21510, Jordan

Correct: Department of General and Special Surgery, Faculty of Medicine, Hashemite University, Zarqa, Jordan.

The original article has been corrected.

## References

[1] Al-Balas (2020). BMC Med Educ.

